# Confusing combination of thrombocytopenia and thrombosis after heparin therapy in a patient with cardiac leiomyosarcoma; a nephrologist viewpoint

**Published:** 2014-07-01

**Authors:** Mohammad-Reza Ardalan

**Affiliations:** ^1^Chronic Kidney Disease Research Center, Tabriz University of Medical Sciences, Tabriz, Iran

**Keywords:** Low molecular weight heparin, Heparin, Thrombocytopenia, Leiomyosarcoma, Thrombosis

## Abstract

We represent here a 30-year-old woman with cardiac leiomyosarcoma who developed thrombocytopenia and deep vein thrombosis a few days after the start of low molecular weight heparin. Thrombocytopenia improved after replacement of low molecular weight heparin with hirudine. We speculated that cardiac leiomyosarcoma is a predisposing factor for development of heparin induced thrombocytopenia.

**Implication for health policy/practice/research/medical education:**

It is possible that cardiac leiomyosarcoma is a predisposing factor for development of heparin induced thrombocytopenia.

## Introduction

Patients with solid tumors are at increased risk of hemostatic imbalance and hypercoagulability (1). Here we report a case of cardiac leiomyosarcoma who developed thrombocytopenia and deep vein thrombosis a few days after the start of heparin.

## Case Presentation

A 30-year-old woman was admitted to our hospital because of exertional dyspnea and chest pain. With suspicion of pulmonary thromboembolism, enoxoparin (a low molecular weight heparin; LMWH) was started. On admission, laboratory examination revealed; white blood cell count (WBC): 11000/μL, serum creatinine; 1 mg/dl and BUN of 35 mg/dl (Table 1).

**Table 1:** Course of laboratory data from the first dose of LMWH first day of admission

**Admission **	**Hemoglobin **	**Platelet** ^*^	**PT** ^**^ ****	**Bilirubin** ^¥^	**AST/ALT** ^¥¥^ ****	**LDH** ^§^
Day						
0 Ŝ	14.1	163000	12 ‏	‏-	‏-	941
3 Đ ‏	13.5 ‏	96000 ‏	‏-	‏-	‏-	‏-
5	13	50000 ‏	17	2.3/1.2	80/85 ‏	1800
6	13	22000 ‏	‏ -			
7	11	24000	18	‏-	‏-	2200
8 Ŧ	11	20000	‏-			
9	‏-	19000 ‏	17.5	3.8/2.0	214/223 ‏	‏-
10 Ħ	11.5	15000 ‏	‏-			
11 Ĩ ‏	‏-	17000 ‏	17.5	5.5/2.5 ‏	319/251 ‏	4500
12 ‏	10.5	25000 ‏	-	‏-	‏-	4200
13		31000	17	‏-		
14		33000	‏-		311/187	‏-
15	12.7	40000	18	‏-	‏-	‏-
19	13.9 ‏	79000 ‏	17	3/1.6 ‏	345/122 ‏	2200
23 † ‏	14 ‏	96000 ‏	18		248 /140 ‏	1800

Ŝ: start of Heparin, Đ: discontinuation of heparin, Ŧ: deep vein thrombosis, Ħ: start of hirudin, Ĩ: IVIG, 0.4 gram/kg, †: surgery and death ^*^µl, ^**^second, ^¥^mg/dl, ^¥¥^IU/ml, ^§^IU/ml

Transthoracic cardiac ultrasound disclosed a 6.2×4 cm mass within the right ventricle, extended to right atrium (RA) and inferior vena cava. Left atrium also had two large mobile masses of 2.52×1.35 and 1.9×1.0 cm in diameter, attached to the mitral valve. Three days after LMWH starting, platelets count dropped to 96000/μl. With suspicion of heparin induced thrombocytopenia (HIT), LMWH was discontinued. At seventh day of admission, left leg became swollen and painful. Doppler study disclosed thrombosis of popliteal and femoral veins with extension to external iliac vein. Treatment was continued with hirudin and immunoglobulin (IVIG 20 g/day/ for five day). Bone marrow aspiration and biopsy disclosed a reactive bone marrow with normal cellularity. Rheumatic factor and antinuclear antibody were negative. D-dimmer; 8 mg/l (<0.3 mg/l), fibrinogen; 70 mg/dl (200-400). Antiphospholipid antibody of IgG and IgM were, 1.6 and 3.7 respectively U/ml (normal<12 U/ml). Also anticardiolipin antibody serum level of IgG and IgM were 5.2 and 9.2 U/ml (normal<12 U/ml) respectively.

Open cardiac surgery was performed on the 23rd day of admission. It revealed a tumoral involvement of right ventricle, left ventricle and inferior vena cava (IVC). Tumor masses had a firm consistency and its pathologic examination was compatible with leiomyosarcoma. Patient finally died because of right ventricular failure and shock state.

## Discussion

The onset of thrombocytopenia and thrombosis a few days after the start of LMWH, and improved of thrombocytopenia after LMWH discontinuation could be compatible with heparin induced thrombocytopenia in this patients. Heparin induced thrombocytopenia usually occurs 5-14 days after heparin start. Its Incidence is 1-5%, and in patients receiving LMWH, its incidence is one

tenth of unfractionated heparin (2); and Eckel et al. described, heparin induced thrombocytopenia, intra-cardiac and IVC thrombosis in a patient with leiomyosarcoma of IVC (3). Thrombocytopenia has been reported in a patient with metastatic leiomyosarcoma of pulmonary vein and left atrium. Stave et al. demonstrated an association between leiomyosarcoma and a possible serum factor that degrades the platelets (4). Antiphospholipid antibody (IgM) was positive in our patient. In our patient and this clinical situation has similarities with antiphospholipid antibody syndrome and infective endocarditic (5-7).

Espiritu et al. have described the occurrence of deep vein thrombosis, IVC thrombosis, positive antiphospholipid antibodies and heparin induced thrombocytopenia in a patient with leiomyosarcoma of IVC (5-7). Elevated serum level of LDH, AST and ALT could be due to microvascular thrombosis and tissue ischemia. Malignant cells contain pro-coagulants and platelet aggregating materials and could be a predisposing factor for development of heparin induced thrombocytopenia (5-7).

## Conclusion

We speculated that heparin may triggers the release of pro-thrombotic and platelet acting materials from cardiac leiomyosarcoma cells, however, this interpretation, should examined in other cases.

## Author’s contribution

MRA is the single author of the paper.

## Conflict of interests

The author declared no competing interests.

## Ethical considerations

Ethical issues (including plagiarism, misconduct, data fabrication, falsification, double publication or submission, redundancy) have been completely observed by the author.

## Funding/Support

None.
